# AMPA Receptors: A Key Piece in the Puzzle of Memory Retrieval

**DOI:** 10.3389/fnhum.2021.729051

**Published:** 2021-09-21

**Authors:** Magdalena Pereyra, Jorge H. Medina

**Affiliations:** ^1^Facultad de Medicina, Universidad de Buenos Aires, Buenos Aires, Argentina; ^2^Instituto de Biología Celular y Neurociencia “Dr. Eduardo De Robertis” (IBCN), CONICET-Universidad de Buenos Aires, Buenos Aires, Argentina; ^3^Instituto Tecnológico de Buenos Aires (ITBA), Buenos Aires, Argentina

**Keywords:** memory retrieval, AMPA receptor, AMPA receptor trafficking, AMPA receptor subunits, memory retrieval mechanisms

## Abstract

Retrieval constitutes a highly regulated and dynamic phase in memory processing. Its rapid temporal scales require a coordinated molecular chain of events at the synaptic level that support transient memory trace reactivation. AMPA receptors (AMPAR) drive the majority of excitatory transmission in the brain and its dynamic features match the singular fast timescales of memory retrieval. Here we provide a review on AMPAR contribution to memory retrieval regarding its dynamic movements along the synaptic compartments, its changes in receptor number and subunit composition that take place in activity dependent processes associated with retrieval. We highlight on the differential regulations exerted by AMPAR subunits in plasticity processes and its impact on memory recall.

## Introduction

The ability to recall past events is a major determinant of survival strategies in all species and is of paramount importance in determining our uniqueness as individuals. Memory retrieval refers to the complex and active process of re-accessing previously stored information and its expression in the brain. Retrieval is critical for memory: without its retrieval it is not possible to ensure we have a given memory. Furthermore, some reports have revealed that some types of amnesia are associated with memory retrieval rather than consolidation failures (Weiskrantz, 1966; Warrington and Weiskrantz, 1968; Roy et al., 2016). Besides, retrieval constitutes a “gateway” process: it could involve memory trace destabilization (Nader et al., 2000b) that can lead to memory reconsolidation or extinction, two critical stages in memory processing (Nader et al., 2000a; Dudai and Eisenberg, 2004; Tronson and Taylor, 2007; Monfils et al., 2009; Kida, 2020). Whether or not memory destabilization takes place depends on the strength and age of the memory as well as the duration of memory retrieval (Suzuki et al., 2004). Even more, retrieval can trigger forgetting of other memory traces that competes with the one being retrieved in a process called “retrieval-induced forgetting” (RIF) (Anderson, 2003; Bekinschtein et al., 2018; Anderson and Hulbert, 2021). For example, in rats, retrieved memories of an object leads to a reduced retention of other objects seen in the same context (Bekinschtein et al., 2018). In humans, some theories point that RIF could be linked to inhibitory mechanisms that reduce the accessibility of non-target items that interfere with the retrieval of target items whereas other theories point to strength-based competition or blocking (Murayama et al., 2014).

Memory recall is a very rapid process, since animals retrieve as soon as they receive, usually without notice, the conditioned stimulus (CS) or other cues, including stimuli remindful of the unconditioned stimulus (US) or an emotional context reminiscent of the memory. Indeed, data from human declarative memory studies indicates that the reinstatement of the patterns of activity needed to retrieve is achieved between 500 and 1500 ms (Staresina and Wimber, 2019). This is clearly a very short timescale compared to other memory processes that could take from minutes to hours (synaptic consolidation), to days or even weeks like systems consolidation (Dudai and Morris, 2000; Squire et al., 2015). Strikingly, there are circumstances in which memory retrieval neural circuit activation could occur without the behavioral output associated, leading to the distinction between memory retrieval and memory expression (Delorenzi et al., 2014).

Memory retrieval involves the reactivation of a relevant number of synapses between learning-activated neurons in various regions of the brain. There is a general consensus that the neuronal activity and synapses that are reactivated when the animals are demanded to retrieve are those that have been changed through the molecular processes that underlie memory formation (Frankland et al., 2019). For those who are interested in what is known about the link between memory formation and retrieval, the multiplicity of brain regions involved in memory retrieval, the interactions between putative “engram cells” and retrieval cues, the neural factors that determine retrieval occurrence, and the consequences of recalling, there are several good review articles dedicated to various aspects of the neurobiology of memory retrieval (Izquierdo et al., 2006; Tronson and Taylor, 2007; Tonegawa et al., 2015; Frankland et al., 2019; Josselyn and Tonegawa, 2020).

Retrieval cues lead to partial reinstatement of the original pattern of neural activity elicited at the moment of encoding (Rugg et al., 2015). In this sense, the overlap between encoding and retrieval neural representations has been proposed in both the transfer-appropriate processing (TAP) and cortical reinstatement hypothesis (Rugg et al., 2008). In particular, theta rhythm has been proposed to set the dynamics for encoding and retrieval within cortical circuits although different phases of hippocampal theta rhythm may enable separation between encoding and retrieval (Hasselmo and Stern, 2014).

In contrast to memory formation, the information about the molecular mechanisms of memory retrieval is surprisingly scarce and fragmentary. In this context, the notion that retrieval constitutes a process as active and involving molecular pathways as intricate as other memory phases is new. In this regard, recent work has revealed the requirement of protein synthesis during memory retrieval (Lopez et al., 2015; Pereyra et al., 2018). Since then, fundamental questions have emerged: Which are the synaptic plasticity proteins whose expression is necessary at the time of retrieval?

The molecular mechanisms of memory retrieval take place in systems that are “ready to go” whenever the animal is demanded to retrieve using pre-existing house-keeping molecules, such as receptors or signaling enzymes. In this review, we will focus on the analysis of some of the molecular mechanisms of memory recall and in particular, we highlight the role of different AMPA receptor (AMPAR) subunits in retrieval of stored information.

## Memory Retrieval: An AMPAR Story

The most prevalent neurotransmitter in the brain is glutamate, which mostly activates AMPAR. AMPAR consists of four homologous pore-forming subunits (GluA1-4) that generally assemble into heteromers. The presence of GluA2 is of functional importance because it confers calcium impermeability to the AMPAR channel (Isaac et al., 2007). In the CA1 area of the hippocampus, GluA1/GluA2 and GluA2/GluA3 heteromers represent approximately 80 and 20%, respectively of the postsynaptic response in baseline conditions (Buonarati et al., 2019).

Memory retrieval has been historically associated with changes in AMPAR. In particular, the first works that studied AMPAR role in memory retrieval with pharmacological approaches have addressed AMPAR activation (Table 1; Liang, 1991; Bianchin et al., 1993; Izquierdo et al., 1993, 1997; Kim et al., 1993; Riedel et al., 1999; Szapiro et al., 2000; Yasoshima et al., 2000). Interestingly, these receptors have a very peculiar fast temporal kinetics which lead to a rapid activation that could support recall processes’ brief timescales. Compared to NMDAR, AMPAR are not blocked by Mg2+ and thus they need less depolarization to be activated (Stern and Alberini, 2013). Indeed, AMPAR activation is needed for NMDAR activation.

**TABLE 1:** Link between AMPAR changes and memory retrieval reported in the literature.

**S. no.**	**AMPAR feature engaged in memory retrieval**	**Animal model**	**Approach**	**Task**	**Brain area**	**References**
1	AMPAR activation	Rat	Pharmacological	Inhibitory avoidance	Amygdala	Liang, 1991
2	AMPAR activation	Rat	Pharmacological	Fear-potentiated startle	Amygdala	Kim et al., 1993
3	AMPAR activation	Rat	Pharmacological	Inhibitory avoidance and habituation to a novel environment	Hippocampus and amygdala	Bianchin et al., 1993
4	AMPAR activation	Rat	Pharmacological	Inhibitory avoidance and habituation to a novel environment	Hippocampus and amygdala	Izquierdo et al., 1993
5	AMPAR activation	Rat	Pharmacological	Inhibitory avoidance	Hippocampus, amygdala, entorhinal cortex, parietal cortex	Izquierdo et al., 1997
6	AMPAR activation	Rat	Pharmacological	Open-field water maze	Hippocampus	Riedel et al., 1999
7	AMPAR activation	Rat	Pharmacological	Inhibitory avoidance	Hippocampus	Szapiro et al., 2000
8	AMPAR activation	Rat	Pharmacological	Conditioned taste aversion	Basolateral amygdala	Yasoshima et al., 2000
9	AMPAR activation	Rat	Pharmacological	Paired associated learning (food and its spatial location)	Hippocampus	Day et al., 2003
10	AMPAR activation	Rat	Pharmacological	Spontaneous object recognition	Perirhinal cortex	Winters and Bussey, 2005
11	AMPAR activation	Rat	Pharmacological	Auditory fear conditioning	Basolateral, amygdala	Mamou et al., 2006
12	AMPAR endocytosis	Rat	Pharmacological	Object recognition	Perirhinal cortex	Cazakoff and Howland, 2011
13	AMPAR endocytosis	Mice	Biochemical, pharmacological	Contextual fear conditioning	Hippocampus	Rao-Ruiz et al., 2011
14	AMPAR activation	Rat	Pharmacological	Conditioned taste aversion	Amygdala	Rodriguez-Ortiz et al., 2012
15	Exchange of calcium impermeable to calcium-permeable AMPAR	Rat	Pharmacological, electrophysiological	Auditory cue fear conditioning	Lateral amygdala	Hong et al., 2013
16	AMPAR activation	Rat	Pharmacological	Conditioned taste aversion	Basolateral amygdala	Garcia-delaTorre et al., 2014
17	AMPAR trafficking	Rat	Pharmacological	Auditory fear conditioning	Amygdala	Lopez et al., 2015
18	AMPAR activation	Rat	Pharmacological	Conditioned taste aversion	Insular cortex and amygdala	Osorio-Gómez et al., 2017
19	AMPAR endocytosis	Rat	Pharmacological	Morris water maze	Hippocampus	Wang et al., 2017
20	GluA2-containing AMPAR endocytosis	Rat	Pharmacological	Contextual fear conditioning	Amygdala	Ferrara et al., 2019
21	GluA2-containing AMPAR endocytosis and AMPAR expression	Rat	Pharmacological	Morphine conditioned place preference	Ventromedial prefrontal cortex	Sun et al., 2019
22	S845 GluA1 AMPAR phosphorylation	Mice	Genetic engineering	Contextual fear conditioning, social recognition	Hippocampus	Hasegawa et al., 2019
23	Calcium-permeable AMPAR activity	Rat	Pharmacological	Auditory fear conditioning, contextual fear conditioning	Basolateral amygdala and hippocampus	Torquatto et al., 2019
24	AMPAR endocytosis	Rat	Pharmacological	Morris water maze	Hippocampus	Ashourpour et al., 2020
25	AMPAR expression	Rat	Pharmacological	Contextual fear conditioning	Basolateral amygdala	Guo et al., 2020
26	AMPAR expression and endocytosis	Rat	Pharmacological	Inhibitory avoidance	Hippocampus	Pereyra et al., 2021
27	AMPAR-Gria2 transcription	Mice	Monosynaptic tracing, electrophysiology, immunochemistry, and optogenetics	Two choice spatial discrimination	Hippocampus (DG)	Li et al., 2021
28	AMPAR trafficking	Mice	Freeze fracture replica immunolabeling	Auditory fear conditioning	Amygdala	Seewald et al., 2021

Our thinking on AMPAR role on synaptic plasticity have been traditionally focused on receptor activity and integrity. However, new technical approaches development has expanded this historical view. In the last three decades, findings coming from advances in labeling postsynaptic surface components (Fujimoto, 1995; Masugi-Tokita et al., 2007) as well as single molecule detection has provided enough evidence about AMPAR mobility between different synaptic compartments and its implications in learning and memory processes (Choquet and Triller, 2013; Penn et al., 2017; Zhang et al., 2018). Also, once inserted in the postsynaptic density (PSD) membrane, AMPAR exhibits a singular clustered aggregation in nanodomains in precise alignment with presynaptic sites that account for efficient synaptic transmission (MacGillavry et al., 2013; Nair et al., 2013). The fact that AMPAR could be exchanged between intra and extrasynaptic compartments (Borgdorff and Choquet, 2002; Petrini et al., 2009; Choquet, 2018) in a fast way made these receptors excellent candidates to underlie the processes involved in memory retrieval that may occur in seconds timescale. Indeed, AMPAR resides as the most mobile among all receptors (Borgdorff and Choquet, 2002). Moreover, modulating AMPAR surface diffusion has been shown to restore memory expression in Huntington Disease model (Zhang et al., 2018).

In the last few years, the focus of AMPAR research has shifted from its mere activation toward its dynamic movements between synapse compartments. A special emphasis has been directed to the role of AMPAR endocytosis (Carroll et al., 2001) in the postsynapse where most surface AMPAR resides (Table 1; Cazakoff and Howland, 2011; Rao-Ruiz et al., 2011; Wang et al., 2017; Ferrara et al., 2019; Sun et al., 2019; Ashourpour et al., 2020; Pereyra et al., 2021). AMPAR activity-dependent trafficking allows subtle changes in number and localization of these postsynaptic receptors, which in turn shapes neural plasticity processes. Since the relatively low number of AMPAR in spines, even a mild alteration in AMPAR internalization could have a great impact on neuronal homeostasis and transmission and hence in behavior (Matsuzaki et al., 2001; Tanaka et al., 2005).

Other important AMPAR features concern its subunit composition. AMPARs consist of four homologous pore-forming subunits (GluA1-4) that generally assemble into heteromers (Rossmann et al., 2011). Different GluA subunits are related with distinctive properties to AMPAR. The presence of GluA2 is of functional importance because it confers calcium impermeability to the AMPAR channel (Isaac et al., 2007). Most AMPARs described in the brain contain GluA2 subunit (Wenthold et al., 1996; Sans et al., 2003; Lu et al., 2009; Rozov et al., 2012). Nevertheless, a small group of AMPAR lacking GluA2 or lacking GluA2 specific editing, have been described as calcium permeable (CP) AMPAR (Sommer et al., 1991; Jonas et al., 1994; Brusa et al., 1995). CP-AMPARs are associated with greater single-channel conductance, faster decay kinetics, and an inwardly rectifying biophysical profile (Liu and Cull-Candy, 2000; Nissen et al., 2010) which make them ideal candidates to account for acute synaptic potentiation. In turn, calcium-impermeable AMPARs (CI-AMPARs) which contains GluA2 subunit are involved in basal synaptic transmission and are more stable at the synapse due to GluA2 subunit interaction with synaptic proteins that promotes the retention of the AMPAR in the membrane (Dong et al., 1997; Nishimune et al., 1998; Shi et al., 2001; Pozo et al., 2012). Consistently, GluA2 subunit has been reported to stabilize dendritic spines (Passafaro et al., 2003; Saglietti et al., 2007). In line with these stability properties differences between CI and CP AMPAR, it has been shown that memory retrieval and its LTP associated processes induce a rapid exchange of CI AMPAR to CP AMPAR (Shi et al., 2001; Hong et al., 2013). This exchange is known to occur through endocytosis involving the C-terminal tail of GluA2 (Collingridge et al., 2010).

AMPA receptors number, composition and mobility are related to the two major forms of plasticity that underlie memory, long-term potentiation (LTP) and long-term depression (LTD). LTP induces recruitment of AMPAR while LTD is accompanied by internalization of AMPAR (Yuste and Bonhoeffer, 2001; Malinow and Malenka, 2002; Matsuzaki et al., 2004). In this regard, the necessity and sufficiency of GluA1 and GluA2 C-terminal domain for LTP and LTD, respectively with differential implications on spatial and contextual learning and memory has been recently reported (Zhou et al., 2018) even if discrepancies regarding the requirement of C-terminal domain of GluA1 for LTP were reported by other authors (see Díaz-Alonso et al., 2020). GluA1 trafficking is strongly associated with LTP (Lee et al., 2003; Makino and Malinow, 2009; Huganir and Nicoll, 2013) while GluA2 plays a prominent role in LTD (Diering and Huganir, 2018). It has been shown that optical LTD stimulation can impair memory recall in lateral amygdala whereas LTP stimulation delivery can restore the recall of memory, respectively (Nabavi et al., 2014). Moreover, KIBRA, a gene related with human memory performance has been shown to regulate AMPAR trafficking potentially by affecting LTP and LTD processes (Makuch et al., 2011).

## Memory Retrieval and AMPAR Subunits: A Matter of Timing

**FIGURE 1:**
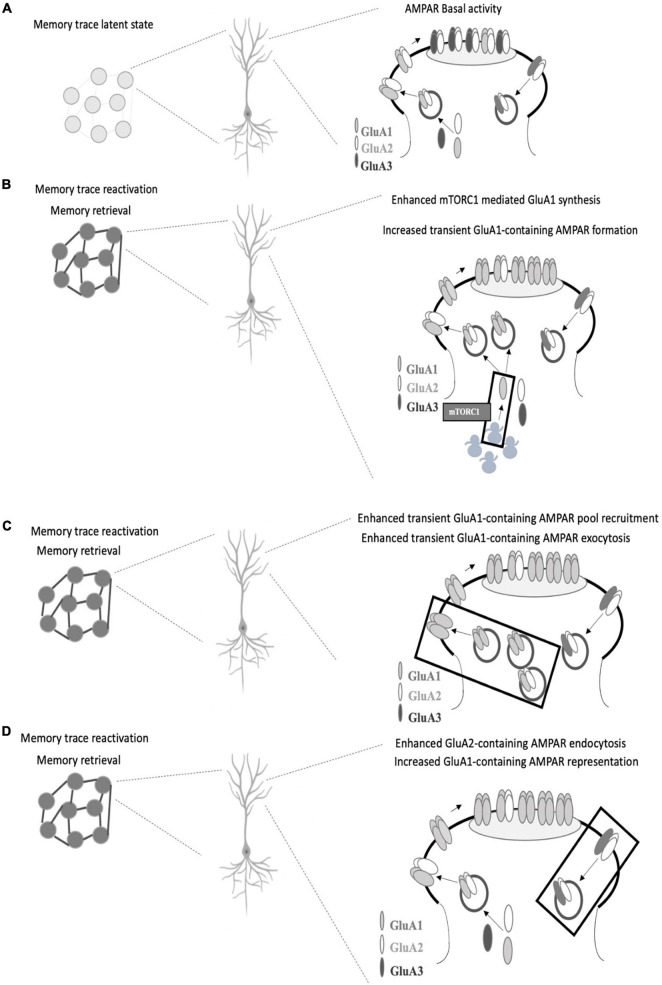
Proposed AMPAR events associated with memory retrieval. **(A)** In a memory trace latent state, GluA1-containing AMPAR are not enough represented at the postsynaptic density and memory recall does not take place. **(B)** GluA1 subunit synthesis is needed for memory retrieval to occur (mediated by mTORC1 pathway as we previously reported), which could favor transient GluA1-containing AMPAR formation (especially GluA1 homomers AMPAR) and surface expression which replaces GluA2-containing AMPAR in the postsynaptic density. **(C)** Alternatively, enhanced GluA1-containing exocytosis may occur in response to activity regulated GluA1-containing AMPAR recruitment. **(D)** Regulated levels of GluA2-containing AMPAR endocytosis may also account for a greater representation of GluA1-containing AMPAR at the synapse. In all the cases, the proposed AMPAR subunit point alterations lead to GluA2 to GluA1 subunit composition shift during memory retrieval. GluA2 synthesis is also required for memory retrieval although GluA2-containing AMPAR seem to have a support role at the time of retrieval.

Neuron ensembles recruited by learning in the CA1 area of the hippocampus exhibit key synaptic changes such as AMPAR insertion (Whitlock et al., 2006). Among AMPAR subunits, GluA1 is delivered in a very fast mode compared to GluA2 and GluA3 upon field stimulation (Tanaka and Hirano, 2012). Considering the similar neural activation pattern between encoding and retrieval and given the fast temporal course of memory retrieval, it is logical to think that GluA1 insertion (Figure 1A) could be considered a critical step for memory retrieval. In this regard, there is a consensus on the need for stable GluA1 levels at the synapse at the time of retrieval (Lopez et al., 2015; Pereyra et al., 2021). Whether these stable levels are achieved by ongoing GluA1 protein synthesis (Lopez et al., 2015; Pereyra et al., 2021) and/or if they are a result of GluA1-AMPAR trafficking (Lopez et al., 2015) or different regulations on GluA1-trafficking, such as phosphorylation of GluA1 on Ser 845 by PKA (Man et al., 2007; Hasegawa et al., 2019) or exchange between CI to CP AMPAR (Hong et al., 2013; Torquatto et al., 2019) remains to be elucidated (Figures 1B,C). Moreover, a recent report has demonstrated that Gria2 (the gene that encodes for GluA2 subunit) transcription reduced the efficacy of memory retrieval, likely by promoting a genetic switch from CP to CI-AMPAR (Li et al., 2021). To dissect the role of GluA1-containing AMPAR synthesis and insertion in memory retrieval (Figures 1B,C), it would be useful to use GluR1_CT_, an interference peptide that selectively disrupts GluA1-containing AMPAR exocytosis (Yu et al., 2008; Cui et al., 2011).

We have demonstrated the need of GluA2 subunit ongoing synthesis during memory retrieval (Pereyra et al., 2021). Whether these GluA2 subunits form GluA2-containing AMPAR to be inserted at the moment of retrieval or if they are needed right after memory retrieval to replenish appropriate levels of GluA2-containing AMPAR at the synapse remains to be further elucidated. In any case, it seems logical that GluA2-containing AMPAR have a supporting rather than a plasticity drive role at the time of retrieval. Various reports indicate that the infusion of Tat-GluR23y (a selective GluA2-containing AMPAR endocytosis blocker) rescued several memory retrieval deficits (Lopez et al., 2015; Pereyra et al., 2021) (Figure 1D). The memory retrieval rescue effect induced by preventing GluA2-containing AMPAR removal remains to be further evaluated. Are these extra retained GluA2-containing AMPAR acting as a reserve pool that then move along the membrane to be recruited in the PSD? Or do they contribute to maintaining a balance between different AMPAR subtypes or between AMPAR across different synaptic compartments? Also, the contribution of GluA3-containing AMPAR to plasticity has been less explored (Kessels and Malinow, 2009; Schwenk et al., 2014; Renner et al., 2017) and a differential role evaluation of GluA1/2 and GluA2/3 in memory retrieval has not yet been addressed.

Among the potential upstream modulators of AMPAR movements at the synapse, mTORC1 has been proposed to play a pivotal role in memory processes (Bekinschtein et al., 2007; Lopez et al., 2015; Pereyra et al., 2021). mTORC1 regulates local protein translation in dendrites (Tang et al., 2002; Hay and Sonenberg, 2004; Hoeffer and Klann, 2010; Raab-Graham and Niere, 2017) and its blockade is associated with downregulation of GluA1 levels during memory retrieval in the PSD (Lopez et al., 2015; Pereyra et al., 2021) (Figure 1B). In the hippocampus, but not in the amygdala, downregulation of GluA1 levels is also observed in the synaptic plasma membrane fraction in rapamycin (selective mTORC1 inhibitor)-treated animals (Lopez et al., 2015; Pereyra et al., 2021). Infusion of Tat-GluR23y rescued mTORC1 blockade effects on memory retrieval while showing no effect on memory retrieval alone (Lopez et al., 2015; Pereyra et al., 2021). Nevertheless, a work has shown that infusion of Tat-GluR23y in the perirhinal cortex impaired object recognition memory retrieval (Cazakoff and Howland, 2011). Further questions in this direction are still unsolved: are AMPAR-mediated retrieval mechanisms brain region and/or memory valence specific? mTORC1 local control of AMPAR subunit translation seems compatible on a temporal scale with the memory retrieval molecular events that occur in the synapse and could provide a rapid synthesis rate that assures AMPAR pools that could be recruited in an activity dependent way.

Another factor to take into account when analyzing AMPAR exchanges lies in the stability of the tetramers once inserted in the membrane. A study that uses single molecule imaging has reported that AMPAR membrane tetramers would be metastable complexes that are in dynamic equilibrium with their respective monomers and dimers (Morise et al., 2019). This would imply dissociations of the AMPARs that allow changes in subunit composition at a very fast rate. Finally, different characteristics and regulatory mechanisms of the different AMPAR subunits (Hirano, 2018) can influence the assembly, insertion, lateral diffusion, and endocytosis rate of AMPAR and thus the associated molecular events that account for memory retrieval.

It is also interesting to evaluate the different AMPAR subunits role in some types of memory forgetting that usually represents a reversible memory retrieval disruption (Li et al., 2014; Guskjolen, 2016; Roy et al., 2016). For example, GluA2-containing AMPAR endocytosis mediates memory forgetting (Hardt et al., 2014; Dong et al., 2015; Migues et al., 2016) while blocking this phenomenon reverses memory retrieval deficits (Lopez et al., 2015; Pereyra et al., 2021). Besides, GluA2-containing AMPAR removal has been proposed to contribute to neuron excitability decrease inducing forgetting (Frankland et al., 2013).

## Concluding Remarks

In this review, we have mainly focused on findings coming from rodent studies. Understanding the molecular basis of memory retrieval is extremely important to fulfill the gap between basic and clinical research. Potential clinical applications include the development of new or more precise targets for the treatment of human memory retrieval pathologies or dysfunction.

Memory recall represents an attractive therapeutic time window for potential translational interventions. Retrieval enables both memory updating and weakening. In this regard, involuntary memory retrieval of a traumatic event is one hallmark symptom of posttraumatic stress disorder (American Psychiatric Association, 2013). Also, memory retrieval deficit has been reported in early stages of Alzheimer mouse model (Roy et al., 2016). On the other hand, there are few reports that address memory retrieval enhancement (Izquierdo et al., 2001, 2003; Barros et al., 2002, 2003; Stern and Alberini, 2013).

Regarding potential memory retrieval enhancement, ampakines, small molecules that positively regulate AMPAR, have been widely evaluated in a variety of rodent models and human studies as a therapeutic avenue for treating memory disorders and to enhance cognitive function (Lynch and Gall, 2006; Lynch et al., 2011, 2014; Seese et al., 2020). For instance, CX-691, a specific ampakine, has been shown to improve memory in a rodent model of Alzheimer (Mozafari et al., 2018). In humans, CX-691 has been shown to acutely improve short-term memory in healthy elderly volunteers (Wezenberg et al., 2007). Ampakine S18986 has shown a memory-enhancing effect on performance and reversed memory impairment induced by aging in a contextual and serial discrimination task that serves as model of declarative memory in mice (Tronche et al., 2010).

Further comprehension on AMPAR-mediated plasticity mechanisms will shed light on more selective potential targets (Zhang and Bramham, 2020) regarding memory retrieval deficits associated with neurological diseases.

## Author Contributions

MP and JHM conceived the content. MP performed bibliography research and wrote the manuscript. JHM organized and corrected the manuscript. Both authors contributed to the article and approved the submitted version.

## Conflict of Interest

The authors declare that the research was conducted in the absence of any commercial or financial relationships that could be construed as a potential conflict of interest.

## Publisher’s Note

All claims expressed in this article are solely those of the authors and do not necessarily represent those of their affiliated organizations, or those of the publisher, the editors and the reviewers. Any product that may be evaluated in this article, or claim that may be made by its manufacturer, is not guaranteed or endorsed by the publisher.
